# Depression Relapse during Long-Term Remission due to Media-Amplified Fear during the COVID-19 Pandemic

**DOI:** 10.1155/2021/5682611

**Published:** 2021-10-07

**Authors:** Nobutaka Ayani, Teruyuki Matsuoka, Sumihiro Yamano, Jin Narumoto

**Affiliations:** ^1^Department of Psychiatry, National Hospital Organization, Maizuru Medical Center, 2410 Yukinaga, Maizuru, Kyoto 625-8502, Japan; ^2^Department of Psychiatry, Graduate School of Medical Science, Kyoto Prefectural University of Medicine, 465 Kajii-cho, Kawaramachi-Hirokoji, Kamigyo-ku, Kyoto 602-8566, Japan

## Abstract

The coronavirus disease 2019 (COVID-19) pandemic has reoriented societies across the world and placed a significant burden on caring for mental health among its population. In this study, we reported two cases where patients experiencing severe depression with delusions of having COVID-19 required inpatient treatment after long-term remission owing to the negative impact of media reports related to the pandemic. Despite the aggravation of their anxiety, the patients were unable to distance themselves from negative information in attempts to remain informed through media to prevent their families and themselves from being infected. Self-protection through improved media literacy is imperative for people to protect themselves from the fearmongering of the media and infodemic in the present-day scenario.

## 1. Introduction

The coronavirus disease 2019 (COVID-19) pandemic has resulted in more than 200 million confirmed cases and four million deaths globally as of 19 August 2021 [1], transforming societies and causing significant mental health burden. During the pandemic, the prevalence of anxiety and depression among the general population was measured at approximately 30% [2]. Notable are the factors associated with mental health disorders included coping mechanisms, as well as exposure to negative news and information from social media [3]. We reported two cases of patients who experienced severe depression with delusions of having COVID-19 and required inpatient treatment after long-term remission in depression owing to the negative impact of media reports related to COVID-19. These cases were diagnosed according to the Diagnostic and Statistical Manual of Mental Disorders, fifth edition (DSM-5) [4] and treated according to the standard guidelines [5, 6]. The patients consented to the publication of this report, and anonymity was ensured. However, the regulations of the ethics committee dictate that such clinical reports do not require any approval.

## 2. Case Presentation

### 2.1. Case 1

Ms. A, a 64-year-old housewife, had been prone to anxiety since she was young. She worked as a needleworker after graduating from high school, got married at the age of 23 years, and had two children. She continued to work as a part-time worker until the age of 59 and then retired and became a housewife. At the age of 45, she developed severe depression with psychomotor retardation due to empty-nest syndrome and was hospitalized for two months. After several months of outpatient care, she completed treatment and was in remission for almost 20 years. In March 2020, an overwhelming amount of negative information about COVID-19 propagated through TV news, tabloids, and social media, such as pessimistic information about the increasing number of the infected cases and deaths, and warning about infection prevention, led her to believe that she and her family would be infected. Consequently, viewing such negative media reports every day led her to have a depressive mood, loss of appetite, insomnia, decreased concentration, and delusion of being infected with COVID-19. This led her to get admitted to a psychiatric ward in a general hospital. On admission, however, the reverse transcription polymerase chain reaction (RT-PCR) test for COVID-19 returned with a negative result. Subsequently, she was diagnosed with severe depression with psychotic symptoms. Electroconvulsive therapy (ECT) was initiated on day 20 since sertraline (100 mg/day) was ineffective on her and she was in a depressive stupor. Although a mild delirium and transient amnesia were occasionally observed in her after ECT, there were no serious adverse events that required discontinuation of ECT. On day 33, delusions were no longer observed, and on day 54, ECT was completed after eight sessions, followed by maintenance treatment with nortriptyline (100 mg/day). The patient's Hamilton Depression Rating Scale (HAM-D) scores improved from 46 points at the start of ECT to 2 points at the end of ETC. She was discharged on day 62.

### 2.2. Case 2

Ms. B, a 52-year-old office worker, had been prone to anxiety since she was young. She worked as an office worker after graduating junior college, got married at the age of 27, and had two children. She resigned from work after her marriage and lived mainly as a housewife but worked occasionally as a part-time employee. At the age of 48, she developed depression due to work-related stress and quit her job unable to take the immense pressure. She recovered through treatment with fluvoxamine and continued to undergo outpatient treatment for several months. In March 2020, after being in remission for about five years, she was influenced by the negative information about COVID-19, rampantly disseminated by the media, such as symptoms presented by the infected people and warnings about preventing the spread of COVID-19 from the news and tabloids. She began worrying that minor somatic symptoms, such as low fever, sore throat, and fatigue, that she experienced, might have been caused by COVID-19, which gradually led to depressive mood, insomnia, decreased concentration, and agitation. She visited hospitals regularly, fearing that she might have been infected and was spreading the virus around her, despite the negative RT-PCR test results. In September 2020, she quit her job following multiple suicide attempts and was admitted to a psychiatric ward in a general hospital. After admission, she was diagnosed with severe depression with psychotic symptoms and continued to question, “What if people around me get infected with coronavirus?” Escitalopram (10 mg/day) was initiated in place of mirtazapine on day 21 since mirtazapine (45 mg/day) and olanzapine augmentation (10 mg/day) was ineffective. Gradually, her depressive mood and agitation improved, and she was discharged on day 56.

## 3. Discussion

The COVID-19 pandemic has affected the development of psychiatric disorders in various ways, including cases of manic episodes after infection [7], severe nightmares about the disease due to fear of infection [8], catatonia due to lockdown [9], and suicide attempts due to fear of infection [10]. In the two cases used in this study, depression recurred owing to anxiety caused by negative information about COVID-19 obtained through various media such as smartphones, TV news, and tabloid TV shows. Despite the aggravation of their anxiety, the patients were unable to distance themselves from negative information because they sought to remain informed about the precautionary measures needed to prevent themselves and their families from being infected. During the pandemic, being female and married were distinguished as primary risk factors for anxiety [11], and consuming stressful content (e.g., the severity of the outbreak, hospital reports) was associated with depression and negative emotions [12]. Preliminary case reports from India and Bangladesh [13, 14] revealed that patients had committed suicide because they thought they were infected, when in fact, they were not [14].

“Infodemic,” which is defined as “a rapid spread of all types of information concerning a problem such that the solution is made more difficult” [15], was first used by some experts in 2003 when severe acute respiratory syndrome (SARS) became a widespread phenomenon. As reflected in the aforementioned cases, this issue resulted in serious repercussions for people's mental health [15]. Accordingly, self-protection through improved media literacy, which is the ability to access, analyze, and evaluate media in various forms, is imperative for people to ensure that they can adequately protect themselves from the fearmongering of the media and the infodemic prevalent in these recent times.

## Data Availability

Data used to support the findings of this study are included within the article.
